# Diffuse Gastrointestinal Metastasis From Breast Cancer: A Case Report and Literature Review

**DOI:** 10.7759/cureus.63608

**Published:** 2024-07-01

**Authors:** Aniqa Faraz, Sydni Kowalczyk, Mark Hendrixson

**Affiliations:** 1 Internal Medicine, Cumberland Medical Center, Crossville, USA; 2 Oncology, Lincoln Memorial University-DeBusk College of Osteopathic Medicine, Crossville, USA; 3 Oncology, Cumberland Medical Center, Crossville, USA

**Keywords:** metastatic breast disease, gi tract, lymph nodes, cancer, breast cancer

## Abstract

Breast cancer (BC) is one of the most common cancers with rare incidence of possible metastatic disease to the gastrointestinal (GI) tract. Early clinical suspicion is important for a timely referral to gastroenterology and for executing a treatment plan. It is difficult to distinguish primary gastric or colon cancer from metastatic disease, and the diagnosis of metastasis can only be established by pathological and immunohistochemistry analysis. We report an interesting case who had metastatic BC to cervical and axillary lymph nodes and was treated with radiation and endocrine therapy. She remained asymptomatic for years, then was found to have rising tumor markers on regular follow-up visits that led to an extensive workup that was negative for tumor recurrence. Five years after radiation therapy, she developed GI symptoms and was referred for esophagogastroduodenoscopy (EGD) and colonoscopy, revealing extensive GI metastatic disease involving the stomach to the rectum. For a patient with metastatic BC who presents with rising tumor markers or gastric symptoms, it is important to do diagnostic studies to rule out GI metastatic disease when no primary disease is identified in the workup.

## Introduction

Metastatic involvement of the gastrointestinal (GI) tract in breast cancer (BC), although a relatively rare clinical occurrence, presents significant diagnostic and therapeutic challenges. The propensity for BC metastases to manifest years after the initial diagnosis with symptoms that can be easily mistaken for more common GI disorders necessitates a high level of clinical vigilance [1]. Recent literature has further highlighted the diversity in the clinical course of these metastases, ranging from asymptomatic cases to those with severe GI symptoms, thereby complicating the diagnostic process [2]. By documenting this case, we aim to shed light on the atypical metastatic patterns of BC and the importance of maintaining a high index of suspicion, particularly in the context of invasive lobular carcinoma (ILC). ILC is more prone to metastasizing to the GI tract [1, 2]. Herein, we report a case of diffuse GI metastatic disease from BC that was initially asymptomatic, but the patient later developed nausea and vomiting as the first symptom, which led to an extensive GI workup. These nonspecific symptoms are sometimes just attributed to being treatment-related or secondary to other primary GI diseases. We reviewed related literature on this unusual metastatic disease, highlighting its diagnosis, treatment, and prognosis.

## Case presentation

A 72-year-old female with a past medical history of hypertension, hyperlipidemia, and osteoarthritis presented with right-sided neck swelling. She had a strong family history of BC. Her mother was diagnosed with BC at the age of 40, her sister at the age of 69, and her paternal aunt at the age of 80. Her father died of prostate cancer at the age of 68. Physical examination revealed multiple small, enlarged lymph nodes in the right cervical and axillary regions. A neck computed tomography (CT) scan confirmed these physical exam findings, leading to the excision of the right cervical and axillary lymph nodes. Pathology of lymph nodes indicated metastatic adenocarcinoma, strongly positive for estrogen and progesterone receptors and negative for human epidermal growth factor receptor 2 (HER2), suggesting a primary origin in BC. Interestingly, a bilateral breast ultrasound and diagnostic mammogram showed no primary lesion in the breast. Breast magnetic resonance imaging (MRI) revealed multiple abnormal right axillary lymph nodes, the largest measuring 2.7 cm, consistent with metastatic disease. She underwent local radiotherapy for the areas of metastatic disease and systemic endocrine therapy with letrozole for five months with complete response. A subsequent positron emission tomography and CT (PET-CT) scan demonstrated resolution of the right axillary and posterior cervical lymphadenopathy. Subsequently, the plan was to continue letrozole therapy for 10 years.

At an annual follow-up with the oncologist four years later, her tumor marker, cancer antigen (CA) 27-29, was noted to be increased at 48.3, but the patient was completely asymptomatic. She underwent PET-CT; CT neck, chest, abdomen, and pelvis; bone scan, and bilateral mammogram with no findings to demonstrate recurrent disease. About a year later, CA 27-29 was 180, and Ibrance was added to letrozole therapy. She complained of mucositis and fatigue, so Ibrance was discontinued. She was then started on Kisqali and fulvestrant along with continued letrozole. She continued this therapy regimen for almost six months, but the CA 27-29 continued to increase to 338. Then, treatment was switched to Xeloda 1000 mg oral twice daily for two weeks and one week off, and she continued this therapy for a few months.

**Figure 1:**
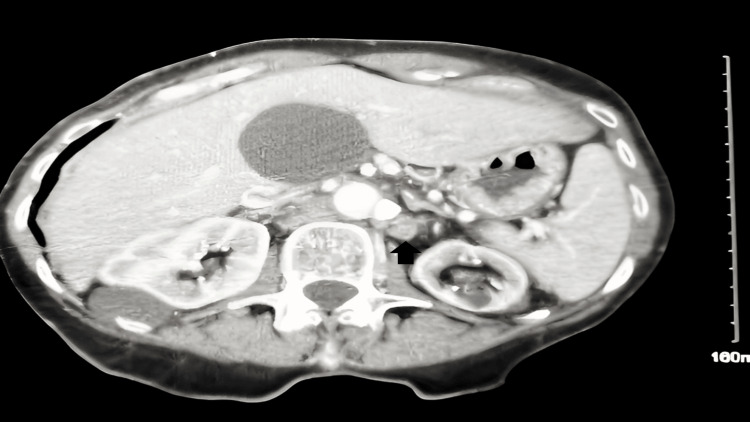
CT abdomen pelvis with IV contrast reveals peri-aortic density on the left that could represent occluded vessel versus adenopathy

She subsequently developed symptoms of decreased appetite, abdominal discomfort, bloating upon eating, and frequent diarrhea. She had nonspecific abdominal tenderness on physical examination. The tumor marker CA 27-29 was 497.1 unit/milliliter (U/mL) (normal range: 38 U/mL or less), and CA 15-3 was elevated to 507.90 U/mL (normal range: 30 U/mL or less). CT imaging of the chest, abdomen, and pelvis with IV contrast at this time revealed retroperitoneal adenopathy in the paraaortic and aortocaval regions, extending bilaterally into the common iliac chains and a left periaortic density potentially indicative of vascular structure or adenopathy (Figure 1).

**Figure 2:**
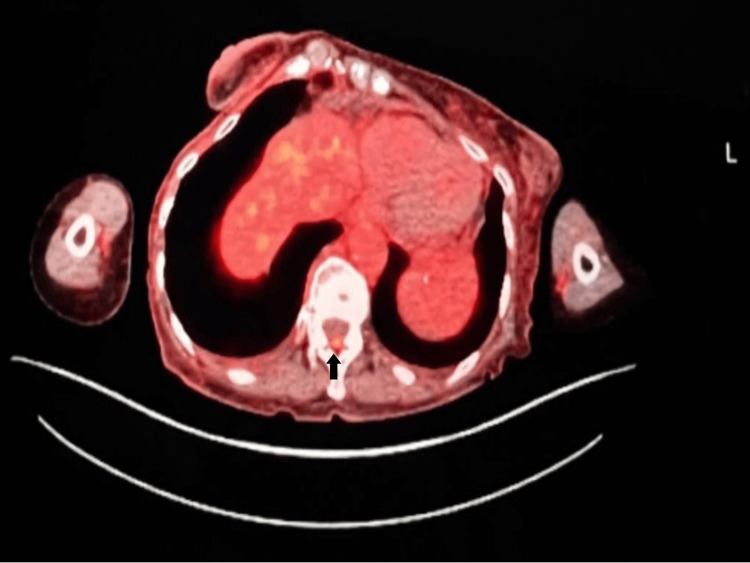
A small focus of abnormal uptake in the T12 posterior epidural space on PET-CT PET-CT: Positron emission tomography–computed tomography.

PET-CT showed activity in the retroperitoneal lymph nodes, left lateral sixth rib, left iliac bone, T12 epidural space, and diffuse nonspecific activity in the colon. A small focus of abnormal uptake in the epidural space at the level of T12 was observed as shown in Figure 2.

**Figure 3:**
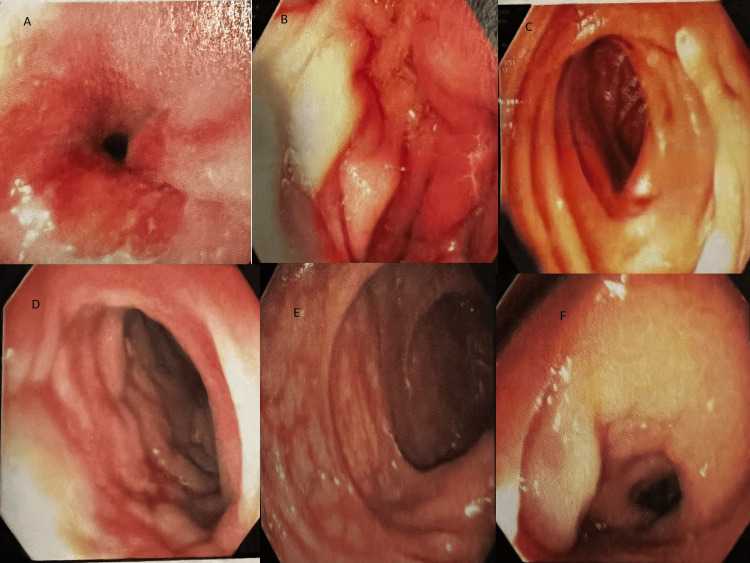
Abnormal mucosa at (A) the lower third of the esophagus: Barrett's esophagus, (B) diffuse atrophic mucosa in the pylorus, (C) lymphangiectasia in the second portion of the duodenum, (D) areas of abnormal mucosa with firm and congested tissue seen in the hepatic flexure, (E) sigmoid colon, and (F) rectum

An esophagogastroduodenoscopy (EGD) and colonoscopy were performed, which identified mucosal changes in the lower third of the esophagus, classified as Barrett's stage C0-M1 (per Prague criteria). The maximum longitudinal extent of these changes was 1 cm, and diffuse atrophic mucosa was seen in the pylorus. Lymphangiectasia was present in the second portion of the duodenum. Areas of congestion and what appeared to be extrinsic compression leading to circumferential stenosis of the colon wall were seen in the hepatic flexure/ascending colon extending to about 8 cm in length, splenic flexure 3 cm in length, and anus to 15 cm upwards (Figure 3).

**Figure 4:**
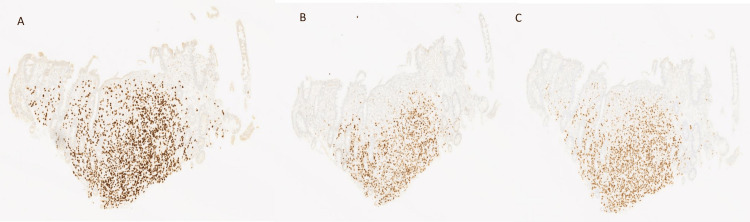
Immunohistochemistry stain showing a nuclear pattern with (A) strong positivity for GATA3 receptors, (B) positivity for estrogen receptors, and (C) positivity for progesterone receptors

Biopsies of the esophagus, gastric body, gastric antrum, duodenum, and ascending colon were taken. Pathological analysis of these biopsies revealed changes suggestive of mild reflux esophagitis with no evidence of malignancy in the esophagus, signet ring cell adenocarcinoma diffusely involving lamina propria in the gastric body, gastric antrum, duodenum, and ascending colon. Immuno-histochemistry was strongly positive for GATA-binding protein 3 (GATA3), estrogen, and progesterone receptors, with HER2 negative, consistent with metastatic BC (Figure 4).

The patient was started on weekly Paclitaxel chemotherapy for three weeks, but she experienced malaise, fatigue, and weakness, so chemotherapy was switched to every other week. Despite this, she showed disease progression with rising tumor markers, so Paclitaxel was discontinued. She was then started on Alpisilib, to which she responded, and her tumor markers started trending down. Her PET fluorodeoxyglucose (FDG) scan did not reveal any abnormal FDG-avid lesions after two months of therapy. Unfortunately, the patient passed away after three months from cancer-related complications.

## Discussion

BC metastasizing to the GI tract is a rare entity ranging from 0.07% to 18%, while in the autopsy series, the range is 8%-35%. The existing literature suggests that it is an atypical site as BC generally spreads to the lymph nodes, bone, liver, lung, and brain [1,3]. The most frequent site for BC metastasis in the GI tract is the stomach (60%), followed by the esophagus (12%), colon (11%), small intestine (8%), rectum (7%), oropharynx (1%), and anus (1%) [4].

These metastatic occurrences are more frequently associated with ILC than invasive ductal carcinoma (IDC). Though the etiology is unclear, some authors reported that it can be related to the tropism of lobular cells. It has been reported that even in mixed histology of primary BC, the lobular subtype metastasizes diffusely to the GI tract, and the rare cases reported with metastatic ductal carcinoma presented only as localized nodular lesions in the stomach. Metastasis to the GI tract can be the first presentation of metastatic BC, or it can present as a relapse of primary cancer after years of diagnosis, as in our case. The small intestine, rectum, oropharynx, and anus involvement are very rare, and oropharyngeal and esophageal metastatic diseases present as dysphagia or pseudoachalasia [4-6]. In our case, the patient had diffuse GI involvement from the stomach to the rectum, which has been reported very rarely.

The clinical manifestations of GI metastases from BC are diverse, encompassing a spectrum from asymptomatic lesions discovered incidentally to GI complaints that could be associated with numerous different conditions [7]. Given their rarity and often nonspecific symptoms, these metastases pose significant diagnostic challenges, sometimes mimicking primary GI disorders like Crohn's disease or primary gastric or colon cancers [8]. The nonspecific presentation underscores the necessity for improved screening and surveillance strategies, particularly in patients with a known history of BC presenting with new GI symptoms. Endoscopic evaluation and biopsy with subsequent immunohistochemical staining are integral to the diagnostic workup as they serve a crucial role in confirming the breast origin of the metastatic lesions [8,9].

After reviewing the literature, the data is limited on the treatment of GI metastasis. Management primarily revolves around systemic therapies tailored to the tumor’s receptor profile, with surgical intervention reserved for specific scenarios such as managing complications of obstructions. The majority of patients are treated with systemic chemotherapy. In some cases, chemotherapy is followed by endocrine therapy as maintenance therapy, and radiation is very rarely used [4,7].

The prognosis for patients with GI metastases from BC remains suboptimal, closely linked to the site and extent of metastatic disease and the hormonal receptor status of the tumor. It is thought to be a pre-terminal event and in cases of gastric metastasis, the survival period was noted to average 8.53 months after diagnosis [10].

## Conclusions

In conclusion, GI metastasis is possible but uncommon, and physicians and oncologists must consider it on the differential even when patients present years later with nonspecific GI symptoms. This case report emphasizes the need for a broad differential diagnosis in breast cancer survivors and reinforces the importance of a comprehensive and multidisciplinary approach to managing these patients. Diagnostic procedures like endoscopic ultrasound-guided biopsy should be performed earlier to obtain a histopathologic diagnosis as it is crucial to distinguish between primary GI disease, primary GI cancer, and metastatic GI disease. Though this disease indicates a poor prognosis, selected patients may benefit from tailored treatment strategies.
